# Maternal and foetal outcomes among 4118 women with HIV infection treated with lopinavir/ritonavir during pregnancy: analysis of population-based surveillance data from the national study of HIV in pregnancy and childhood in the United Kingdom and Ireland

**DOI:** 10.1186/s12879-016-1400-y

**Published:** 2016-02-04

**Authors:** Pat A. Tookey, Claire Thorne, Jean van Wyk, Michael Norton

**Affiliations:** 1 UCL Institute of Child Health, University College London, 30 Guilford St, London, WC1N 1EH UK; 2 AbbVie Inc, 1 North Waukegan Road, North Chicago, IL 60064 USA

**Keywords:** Lopinavir/ritonavir, Pregnancy, HIV, Vertical transmission, Congenital abnormality

## Abstract

**Background:**

The National Study of HIV in Pregnancy and Childhood (NSHPC) conducts comprehensive population-based surveillance of pregnancies in women with HIV infection in the United Kingdom/Ireland. Use of antepartum antiretroviral therapy (ART) for prevention of mother-to-child transmission (MTCT) and to treat maternal infection, if required, is standard practise in this population; lopinavir/ritonavir (LPV/r) is commonly used. The study objective was to examine the use of LPV/r among pregnant women with HIV infection to describe maternal and foetal outcomes.

**Methods:**

The NSHPC study collected maternal, perinatal and paediatric data through confidential and voluntary obstetric and paediatric reporting schemes. Pregnancies reported to the NSHPC by June 2013, due to deliver 2003–2012 and with LPV/r exposure were included in this analysis, using pregnancy as the unit of observation.

**Results:**

Four thousand eight hundred sixty-four LPV/r-exposed pregnancies resulting in 4702 deliveries in 4118 women were identified. Maternal region of birth was primarily sub-Saharan Africa (77 %) or United Kingdom/Ireland (14 %). Median maternal age at conception was 30 years. LPV/r was initiated preconception in 980 (20 %) and postconception in 3884 (80 %) pregnancies; median duration of antepartum LPV/r exposure was 270 and 107 days, respectively. Viral load close to delivery was <50 copies/mL in 73 % and <1000 copies/mL in 94 % of women. 63 % of deliveries were by caesarean section (elective, 62 %; emergency, 38 %). Among singleton live births, 13 % were <37 weeks of gestation (2.5 % <32 weeks) and 15 % had birth weight <2500 g (2.3 % <1500 g). MTCT rates were 1.1 (2003–2007) and 0.5 % (2008–2012). 134 live born children (2.9 %) had ≥1 congenital abnormality.

**Conclusions:**

The results of this analysis using real-world data from a large number of pregnant women with HIV infection in the United Kingdom and Ireland who received LPV/r-containing ART regimens demonstrate that these regimens have a good safety profile and are effective for viral suppression during pregnancy, with associated low rates of MTCT.

## Background

Initially established in 1986 to monitor paediatric patients with AIDS, the United Kingdom and Ireland’s National Study of HIV in Pregnancy and Childhood (NSHPC) has conducted comprehensive population-based surveillance of HIV infection in pregnant women since 1990. Observational data collected through the NSHPC can be used to examine trends in the diagnosis and management of HIV in pregnant women and related pregnancy outcomes. Data from this and other studies have demonstrated that vertical transmission risk reduction strategies, such as the use of antiretroviral therapy (ART) during pregnancy, delivery management and avoidance of breastfeeding, can result in an appreciable reduction of the mother-to-child transmission (MTCT) rate from approximately 20 to 0.5 % among populations living in resource-rich settings [1–8]. In more resource-limited regions, similarly low vertical transmission rates have not yet been achieved, as MTCT prevention efforts still confront numerous challenges, including access to and availability of ART [9–11].

Guidelines issued by the British HIV Association (BHIVA) and the World Health Organization (WHO) for the management of HIV infection in women during pregnancy recommend combination ART for maternal treatment needs and to reduce the risk of MTCT [12–14]. For pregnant women with HIV infection who require ART for their own health, based on CD4+ T-cell count or disease stage, immediate initiation or continuation of ART is advised. National recommendations vary regarding the timing of initiation of ART to reduce the risk of MTCT during pregnancy in women with HIV who do not need ART for their own health [12–15]. In the United Kingdom, current BHIVA guidelines recommend initiating ART by 24 weeks of gestation for this group of women, with a range of factors to be considered in deciding when to begin therapy, including maternal CD4+ T-cell count, baseline viral load and potential for preterm delivery [12]. As with any drug taken during pregnancy, antenatal exposure to ART agents raises concerns regarding toxicity, teratogenicity and an increased risk of adverse pregnancy outcomes, including preterm delivery, low birth weight and congenital abnormalities [16–19].

The incidence of pregnancy in women with HIV infection accessing HIV clinical care substantially increased between 2000 and 2009 in the United Kingdom, and subsequently stabilised. Increasing numbers of women with HIV infection are initiating or maintaining ART during pregnancy [3, 7, 20, 21]. Lopinavir, a protease inhibitor that is co-formulated with ritonavir (LPV/r), is commonly used as an anchor drug as part of an ART regimen during pregnancy. Previous investigations into the association between the use of protease inhibitor–containing ART during pregnancy and preterm delivery have yielded mixed results [18, 22–28]. A 2013 systematic review of 9 studies of ART in pregnant women with HIV infection (*n*=2675, in total) found that LPV/r use was associated with reduced maternal viral load at the time of delivery and MTCT rates <3 %, and identified no unique safety concerns regarding preterm delivery or low birth weight [29]. However, the majority of patients included in this analysis lived in resource-limited regions, limiting the generalisability of the findings to populations living in other settings.

Using data from the NSHPC for pregnancies that were due to deliver between 2003 and 2012, the current study examined the use of LPV/r among pregnant women with HIV infection in the United Kingdom and Ireland to explore its association with maternal and foetal outcomes, including viral suppression, gestational age at delivery, prevalence of low birth weight at delivery, MTCT rates and reported adverse infant outcomes.

## Methods

The NSHPC is a population-based surveillance study of HIV in women and children in the United Kingdom and Ireland that collects maternal, perinatal and paediatric data through 2 parallel, confidential and voluntary reporting schemes: an obstetric scheme for the notification of pregnancies in women with HIV infection and a paediatric scheme through which infants exposed to HIV and children who have HIV are reported; full details are published elsewhere [4]. Pregnancies reported through the obstetric scheme were included in this retrospective analysis of individual patient data if the mother was identified as having a positive test for HIV infection by the time of delivery, had an expected date of delivery between 1 January 2003 and 31 December 2012 and received LPV/r at any time during her pregnancy. LPV/r was used in approximately 37 % of all pregnancies reported to the NSHPC in this period. Pregnancies that did not have a reported outcome by 30 June 2013 were excluded. Pregnancy was used as the unit of observation. HIV testing guidelines called for polymerase chain reaction (PCR) testing shortly after birth, and 2 weeks and 2 months after cessation of infant prophylaxis. Antibody testing at 18 months was also recommended.

### Ethics approval

The National Study of HIV in Pregnancy and Childhood has London Multi-Centre Research Ethics Committee (NHS) approval (MREC/04/2/009). Individual patient consent is not sought or required for reporting to the NSHPC, which is an observational surveillance study subject to review by appropriate national information governance bodies (NIGB Ref: PIAG/BPSU 2–10(a)/2005, IG Toolkit reference: Version 12 2014/15 EE133902-FOPHS-PPAPPIASG; now under review with the Health Research Authority’s Confidentiality Advisory Group). This analysis was based on data already collected through the routine NSHPC study protocols and, per study approvals, individual patient consent or further permissions are not required. Furthermore, no disaggregated data were shared with anyone outside the NSHPC, and the report is totally anonymised.

### Definitions

Maternal age was the mother’s age at conception. Trimesters were defined as follows: first, <13 completed gestational weeks; second, between 13 and 26 completed gestational weeks; and third, ≥27 completed gestational weeks. Low infant birth weight was defined as <2500 g.

Baseline CD4+ T-cell counts and viral loads (HIV RNA measurement reported as copies/mL) were the first reported measurements in the first or second trimester of pregnancy. CD4+ T-cell counts and viral loads at delivery were reported as the measurements taken closest to delivery during the third trimester or within 7 days after delivery. If delivery was preterm with no maternal viral load or CD4+ T-cell count reported in the third trimester, the last maternal measurement taken up to 28 days before and 7 days after delivery was used. Caesarean deliveries were classified as elective if they were performed before the initiation of labour and the rupture of membranes. A delivery was classified as preterm if it occurred before 37 completed gestational weeks. Infant HIV infection status was classified as uninfected or infected on the basis of reported PCR or HIV antibody results, or indeterminate for infants whose infection status had not yet been reported. Viral suppression was defined as an HIV RNA measurement <50 copies/mL.

Congenital abnormalities were classified according to the WHO *International Classification of Diseases*, *10th revision*, using information provided by clinicians at infant notification or at follow-up.

### Statistical analysis

Statistical analysis was performed using STATA v12.0 software (StataCorp, College Station, TX, USA), and standard descriptive statistics were used to summarise the data, with proportions, medians and interquartile ranges (IQRs) presented. Comparisons were assessed using a *χ* ^2^ test for categorical variables; 95 % confidence intervals (CIs) were calculated for rates.

## Results

### Baseline maternal and disease characteristics

**Fig. 1:**
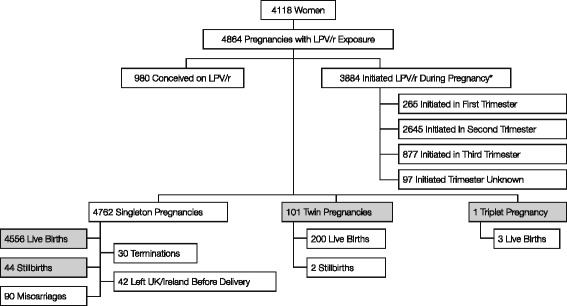
Exposure to LPV/r and pregnancy outcomes in the study population. LPV/r = lopinavir/ritonavir. *Includes pregnancies (*n*=281; 7.2 %) that were conceived whilst the woman was receiving a non-LPV/r-containing regimen. Shading indicates pregnancies that resulted in live births or stillbirths (*n*=4702)

The study population comprised 4118 women with 4864 pregnancies with LPV/r exposure, who were due to deliver between 1 January 2003 and 31 December 2012. These pregnancies resulted in 4759 live births (4556 singletons, 99 sets of twins, 2 single twins and 1 set of triplets), 46 stillbirths (44 singletons and 2 twins), 90 miscarriages and 72 other outcomes (terminations or left the United Kingdom/Ireland before delivery; Fig. 1). The pregnancies were stratified by timing of LPV/r exposure, with 980 pregnancies conceived whilst the woman was receiving LPV/r therapy and 3884 pregnancies whilst LPV/r was initiated in the antenatal period. In most cases, when LPV/r treatment was initiated during pregnancy (3603/3884; 92.8 %), women were not receiving any ART at conception; the other 281 pregnancies were conceived on regimens that did not contain LPV/r and a switch to LPV/r occurred during the antenatal period. Of the 4831 pregnancies for which nucleoside reverse transcriptase inhibitor (NRTI) backbone therapy data were available, zidovudine (ZDV) + lamivudine (3TC), emtricitabine (FTC) + tenofovir (TDF), and abacavir (ABC) + 3TC were the most common, accounting for 85 % (4106/4831) of all backbone therapy regimens administered. When considered as a group, the ZDV + 3TC, FTC + TDF, or ABC + 3TC backbones were administered more frequently in pregnancies with the initiation of LPV/r (88.9 %; 3432/3862) than in pregnancies with conception on LPV/r (69.6 %; 674/969). Of the women with a viral load available at delivery, 73 % (2979/4083) were suppressed overall (Table 1).

**Table 1:** Maternal demographic and disease characteristics, stratified by timing of LPV/r exposure^a^

Variable	All Pregnancies (*N*=4864)	Pregnancies With Conception on LPV/r (*n*=980)	Pregnancies With Initiation of LPV/r (*n*=3884)	*P* Value Between Groups
	*N* (%) or Median (IQR)			
Age at conception, y	30.4 (26.6–34.3)	33.3 (29.8–36.6)	29.7 (26.0–33.6)	<0.001*
Ethnic group				
ᅟWhite	742 (15.3)	135 (13.8)	607 (15.6)	0.07
ᅟBlack African	3742 (77.0)	781 (79.7)	2961 (76.3)	
ᅟOther	377 (7.8)	64 (6.5)	313 (8.1)	
ᅟMissing^b^	3	–	3	
Region of birth				
ᅟUnited Kingdom/Ireland	663 (13.8)	114 (11.8)	549 (14.3)	0.022*
ᅟSub-Saharan Africa	3678 (76.5)	769 (79.3)	2909 (75.8)	
ᅟElsewhere	469 (9.8)	87 (9.0)	382 (9.9)	
ᅟMissing^b^	54	10	44	
Timing of maternal HIV diagnosis				
ᅟPre-pregnancy	2914 (59.9)	980 (100)	1934 (49.8)	–
ᅟDuring pregnancy	1950 (40.1)	–	1950 (50.2)	
ᅟᅟFirst trimester	550 (11.3)	–	550 (14.2)	
ᅟᅟSecond trimester	970 (19.9)	–	970 (25.0)	
ᅟᅟThird trimester	206 (4.2)	–	206 (5.3)	
ᅟᅟTrimester unknown	224 (4.6)	–	224 (5.8)	
HIV symptoms in pregnancy				
ᅟYes	257 (5.7)	99 (11.1)	158 (4.3)	–
ᅟNo	4289 (94.3)	792 (88.9)	3497 (95.7)	
ᅟMissing^b^	318	89	229	
Maternal HIV risk factor				
ᅟNot IDU-related	4784 (98.4)	956 (97.6)	3828 (98.6)	–
ᅟIDU-related	80 (1.6)	24 (2.4)	56 (1.4)	
NRTI backbone				
ᅟZDV + 3TC	3306 (68.0)	271 (27.7)	3035 (78.1)	–
ᅟFTC + TDF	499 (10.3)	247 (25.2)	252 (6.5)	–
ᅟABC + 3TC	301 (6.2)	156 (15.9)	145 (3.7)	–
ᅟOther	725 (14.9)	295 (30.1)	430 (11.1)	–
ᅟMissing	33 (0.7)	11 (1.1)	22 (0.6)	–
Baseline CD4+ T-cell count in pregnancy,^c^ n	3593	752	2841	–
ᅟMissing^b^	1271	228	1043	–
ᅟMedian, cells/mm^3^	390 (270–540)	460 (330–600)	370 (260–520)	<0.001*
Pre-LPV/r initiation CD4+ T-cell count, n	–	N/A	2503	–
ᅟMedian, cells/mm^3^	–	N/A	370 (260–520)	–
ᅟMissing^b^	–	N/A	1381	–
Delivery CD4+ T-cell count,^d^ n	3712	688	3024	–
ᅟMedian, cells/mm^3^	450 (320–590)	440 (330–560)	450 (310–600)	–
ᅟMissing^b^	1152	292	860	–
Viral load at baseline,^c^ n	–	716	3336^e^	–
ᅟMedian, copies/mL	–	N/A	5200 (862–22,279)	–
ᅟ<50 copies/mL	–	587 (82.0)	N/A	–
ᅟ≥50 copies/mL	–	129 (18.0)	N/A	–
ᅟMissing	–	182	910	–
Viral load at delivery,^d^ n	–	747	3336^e^	–
ᅟ<50 copies/mL	–	677 (90.6)	2302 (69.0)	–
ᅟ≥50–999 copies/mL	–	70 (9.4)	798 (23.9)	–
ᅟ≥1000 copies/mL	–	–	236 (7.1)	–
ᅟMissing	–	151	910	–

*Abbreviations*: *LPV/r* lopinavir/ritonavir, *IQR* interquartile range, *IDU* injecting drug use, *ZDV* zidovudine, *3TC* lamivudine, *FTC* emtricitabine, *TDF* tenofovir, *ABC* abacavir, *N/A* not available

*Statistically significant, *P* < 0.05

^a^Maternal characteristics were determined on the basis of pregnancies; therefore, some women appear more than once because of repeat pregnancies reported in the study period

^b^Excluded from denominator

^c^First reported measurements in the first or second trimester of pregnancy

^d^Measurements reported closest to delivery in the third trimester or within 7 days of delivery. If not available, the last measurement taken up to 28 days before and 7 days after delivery was used

^e^Pregnancy where the outcome was a live birth or stillbirth and data were available on the timing of initiation of LPV/r and delivery viral load

As shown in Table 1, median maternal age was 30.4 years (range, 26.6–34.3 years); women who conceived whilst on LPV/r were significantly older than those who began LPV/r antenatally (*P* < 0.001). The majority of pregnancies were in women who were of black African descent (3742/4861; 77.0 %) and/or were born in sub-Saharan Africa (3678/4810; 76.5 %). In 49.8 % of pregnancies (1934/3884) where LPV/r was initiated during pregnancy, HIV had already been diagnosed before conception.

### CD4+ T-cell counts

Baseline CD4+ T-cell counts were available for 3593 of 4864 pregnancies (73.9 %). As shown in Table 1, the baseline median CD4+ T-cell count of 460 cells/mm^3^ (IQR, 330–600 cells/mm^3^) among pregnancies conceived on LPV/r was significantly higher than the median of 370 cells/mm^3^ (IQR, 260–520 cells/mm^3^) observed in pregnancies where LPV/r was initiated antenatally (*P* < 0.001). Among women who initiated LPV/r in pregnancy, CD4+ T-cell count before initiation was available for 2503 of 3884 pregnancies (64.4 %). Median CD4+ T-cell count was 370 cells/mm^3^ (IQR, 260–520 cells/mm^3^); 43.7 % of these women (1093/2503) had a CD4+ T-cell count <350 cells/mm^3^ and 13.3 % (332/2503) had a CD4+ T-cell count <200 cells/mm^3^. CD4+ T-cell counts close to delivery were available for 3712 of 4864 pregnancies (76.3 %). Comparing baseline and delivery CD4+ T-cell counts in those with initiation of LPV/r in pregnancy, there was a significant improvement, with the median increasing from 370 to 450 cells/mm^3^ (*P* < 0.001). The difference between median CD4+ T-cell count at baseline and at delivery among pregnancies conceived on LPV/r was not statistically significant (*P*=0.13). Among all pregnancies with a delivery CD4+ T-cell count available, approximately 40 % (1510/3712) achieved CD4+ T-cell counts ≥500 cells/mm^3^.

### Viral load measurements

For 79.7 % of pregnancies (716/898) with conception on LPV/r, a viral load measurement in the first or second trimester was available (Table 1). In 82.0 % of these pregnancies (587/716), the woman had an undetectable viral load (<50 copies/mL) at baseline. A total of 95.2 % of pregnancies (453/476) with a baseline undetectable viral load maintained a suppressed viral load at delivery (based on pregnancies with data available for both time points). In pregnancies where there was a detectable viral load in the first or second trimester, suppression was achieved by delivery for the majority of women (84/111; 75.7 %).

### Timing and duration of LPV/r exposure

Among 4702 pregnancies resulting in a live birth or a stillbirth, the date of initiation of LPV/r therapy was available for 4609 pregnancies. Of these, 898 pregnancies (19.5 %) had LPV/r exposure from before conception, and the median duration of antenatal LPV/r exposure was 270 days (IQR, 263–277 days). Treatment with LPV/r was usually initiated during the second trimester (2597/4609; 56.3 %); initiation during the first trimester (241/4609; 5.2 %) or third trimester (873/4609; 18.9 %) was less common. Of the 3711 women who initiated therapy in pregnancy for whom initiation dates were available, the median duration of antenatal LPV/r exposure was 107 days (IQR, 79–132 days).

There were 3336 pregnancies with LPV/r initiated after conception, which resulted in live births or stillbirths and for which viral load at delivery and timing of LPV/r initiation were available (Table 1). The median viral load in this group was 5200 copies/mL (IQR, 862–22,279 copies/mL) at baseline. By delivery, 69.0 % of women (2302/3336) had achieved viral suppression, with most of the women in this group beginning LPV/r treatment in the second trimester (1731/2302; 75.2 %). In another 23.9 % of pregnancies (798/3336) in which LPV/r was initiated after conception, a viral load of 50 to 999 copies/mL was achieved by delivery. Among the 7.1 % of pregnancies (236/3336) where the maternal viral load was ≥1000 copies/mL at delivery, the median viral load at the time of delivery was 5083 copies/mL (IQR, 1989–24,900 copies/mL); LPV/r therapy was not initiated until the third trimester in 59.3 % of these pregnancies (140/236).

### Pregnancy outcomes

A total of 95.7 % (4556/4762) of the singleton pregnancies resulted in a live birth, whilst 98.0 % (99/101) of twin pregnancies and a triplet pregnancy resulted in live births. Of singleton births, 63.4 % of the infants (2877/4541) were delivered by elective or emergency caesarean section and 36.6 % of infants (1664/4541) were delivered vaginally (Tables 2 and 3). The median gestational age was 38 weeks (IQR, 38–39 weeks); 12.8 % of the deliveries (585/4556) were pre-term. Of the 101 sets of twins and 1 set of triplets, 50 were delivered by elective caesarean section, 38 by emergency caesarean section and 14 vaginally; 10 deliveries were at <32 weeks gestation, and 47 were at 32–36 completed weeks. There were no significant differences in the proportion of pre-term deliveries among singleton live births and stillbirths according to NRTI backbone (*P*=0.63; Tables 2 and 3). The median birth weight was 3030 g (IQR, 2710–3360 g); 85.3 % of the infants (3749/4395) weighed ≥2500 g and 2.3 % of the infants (101/4395) weighed <1500 g. For the twins and triplets, birth weight was not provided for 7/205 infants. Excluding those for whom birth weight was not available, 8.8 % weighed <1500 g, 46.8 % 1500–2499 g and 41 % ≥2500 g; median birth weight was 2350 g (IQR, 1930–2612 g). Fifteen percent of infants (460/3064; live born and stillborn) weighed <2500 g in pregnancies in which the mother was treated with a ZDV + 3TC backbone, with similar proportions for those with an FTC + TDF backbone (14.9 %; 63/261) and an ABC + 3TC backbone (13.9 %; 38/273; *P*=0.89); no difference by NRTI backbone was observed when restricting to term infants (data not shown). For pregnancies that were delivered at term, an infant mortality rate of 1.5 per 1000 infants (95 % CI, 0.3–2.7) was reported. The percentage of stillbirths was similar between pregnancies conceived on LPV/r and pregnancies where LPV/r was initiated during pregnancy. Both the infant mortality rate and the percentage of stillbirths were inversely correlated with gestational age. A higher percentage of reported miscarriages or terminations were found in the pregnancies conceived while on LPV/r (8.0 %; 76/952) compared with pregnancies during which LPV/r therapy was initiated (1.2 %; 44/3810).

**Table 2:** Outcomes of singleton pregnancies with LPV/r exposure (*n*=4762)

Total live births, *n* (%)	4556 (95.7)
ᅟConception on LPV/r, *n*	863
ᅟInitiation of LPV/r, *n*	3693
Mode of delivery, *n* (%)	*n*=4541
ᅟVaginal delivery	1664 (36.6)
ᅟElective caesarean delivery	1776 (39.1)
ᅟEmergency caesarean delivery	1101 (24.3)
Gestational age	*n*=4556
ᅟMedian (IQR), wk	38 (38–39)
ᅟ<32 wk, *n* (%)	112 (2.5)
ᅟ32–36 wk, *n* (%)	473 (10.4)
ᅟ≥37 wk, *n* (%)	3971 (87.2)
Birth weight	*n*=4395
ᅟMedian (IQR), g	3030 (2710–3360)
ᅟ<1500 g, *n* (%)	101 (2.3)
ᅟ1500–2499 g, *n* (%)	545 (12.4)
ᅟ≥2500 g, *n* (%)	3749 (85.3)
Infant mortality, *n*	24
ᅟRate by gestational age	
ᅟᅟ<32 wk, *n*/N, per 1000 infants (95 % CI)	14/112, 125.0 per 1000 infants (62.8–187.2)
ᅟᅟ32–36 wk, *n*/N, per 1000 infants (95 % CI)	4/473, 8.5 per 1000 infants (0.2–16.7)
ᅟᅟTerm, *n*/N, per 1000 infants (95 % CI)	6/3971, 1.5 per 1000 infants (0.3–2.7)
Stillbirth, *n* (%)	44 (0.9)
ᅟConception on LPV/r, *n*/N (%)	7/952 (0.7)
ᅟInitiation of LPV/r, *n*/N (%)	37/3810 (1.0)
ᅟRate by gestational age	
ᅟᅟ<32 wk, *n*/N, per 1000 infants (95 % CI)	23/135, 170.4 per 1000 infants (106.1–234.6)
ᅟᅟ32–36 wk, *n*/N, per 1000 infants (95 % CI)	8/481, 16.6 per 1000 infants (5.2–28.1)
ᅟᅟTerm, *n*/N, per 1000 infants (95 % CI)	13/3984, 3.3 per 1000 infants (1.5–5.0)
Miscarriage or termination,^a^ *n* (%)	120 (2.5)
ᅟConception on LPV/r, *n*/N (%)	76/952 (8.0)
ᅟInitiation of LPV/r, *n*/N (%)	44/3810 (1.2)
Left United Kingdom/Ireland before delivery, *n* (%)	42 (0.9)

*Abbreviations*: *LPV/r* lopinavir/ritonavir, *IQR* interquartile range

^a^Based on data reported from antenatal care providers

**Table 3:** Outcomes of singleton pregnancies by NRTI backbone (*n* = 4762)

	ZDV + 3TC	FTC + TDF	ABC + 3TC	Other/Missing	*P* value
Preterm, *n* (%)					
ᅟNo (≥37 wk)	2762 (87.0)	380 (86.2)	249 (88.6)	565 (84.0)	0.13
ᅟYes (<37 wk)	412 (13.0)	61 (13.8)	32 (11.4)	108 (16.0)	
Gestational age, *n* (%)					
ᅟ≥37 wk	2762 (87.0)	380 (86.2)	249 (88.6)	565 (84.0)	0.31
ᅟ32–36 wk	317 (10.0)	49 (11.1)	24 (8.5)	88 (13.1)	
ᅟ<32 wk	95 (3.0)	12 (2.7)	8 (2.9)	20 (3.0)	
Low birth weight, *n* (%)					
ᅟNo (≥2500 g)	2604 (85.0)	361 (85.1)	235 (86.1)	536 (84.5)	0.95
ᅟYes (<2500 g)	460 (15.0)	63 (14.9)	38 (13.9)	98 (15.5)	
Low birth weight in term infants, *n* (%)					
ᅟNo (≥2500 g)	2492 (93.1)	340 (92.6)	226 (93.0)	502 (93.0)	0.99
ᅟYes (<2500 g)	184 (6.9)	27 (7.4)	17 (7.0)	38 (7.0)	

*Abbreviations*: *NRTI* nucleoside reverse transcriptase inhibitor, *ZDV* zidovudine, *3TC* lamivudine, *FTC* emtricitabine, *TDF* tenofovir, *ABC* abacavir

### Mother-to-child transmission rates

Data were available at the time of analysis on the HIV infection status for 88.7 % of the singleton infants (4039/4556). In the years 2008–2012, the overall MTCT rate was 0.5 % (12/2406; 95 % CI, 0.2–0.8 %), representing a >50 % decrease from the MTCT rate in the years 2003–2007 (1.1 % [18/1633]; 95 % CI, 0.6–1.6 %). As noted in Table 4, higher MTCT rates were associated with lower baseline maternal CD4+ T-cell counts and later initiation of LPV/r therapy, with MTCT rates of approximately 2 % observed when LPV/r therapy was not initiated until the third trimester, in both reporting periods. Because of reporting delays due to recent birth, data on HIV infection status were not yet available for 517 singleton infants. No infections were reported in the twins or triplets (*n*=176) with known HIV status; 27 of the twins and triplets (13 %) had an indeterminate infection status.

**Table 4:** Stratified MTCT rates in singleton pregnancies^a^

	2003–2007		2008–2012	
	*n*/N	% (95 % CI)	*n*/N	% (95 % CI)
Overall MTCT rate	18/1633	1.1 (0.6–1.6)	12/2406	0.5 (0.2–0.8)
ᅟBy timing of LPV/r initiation				
ᅟᅟBefore conception	2/333	0.6 (0.2–2.2)	2/635	0.3 (0.1–1.1)
ᅟᅟFirst trimester	0/33	–	0/77	–
ᅟᅟSecond trimester	8/858	0.9 (0.5–1.8)	5/1397	0.4 (0.2–0.8)
ᅟᅟThird trimester	8/376	2.1 (1.1–4.1)	5/264	1.9 (0.8–4.4)
ᅟBy baseline CD4+ T-cell count				
ᅟᅟ<200 cells/mm^3^	3/157	1.9 (0.7–5.5)	2/206	1.0 (0.3–3.5)
ᅟᅟ200–349 cells/mm^3^	4/340	1.2 (0.5–3.0)	1/506	0.2 (0.04–1.1)
ᅟᅟ≥350 cells/mm^3^	4/600	0.7 (0.3–1.7)	2/1171	0.2 (0.1–0.6)
ᅟᅟMissing CD4+ T-cell count	7/536	1.3 (0.6–2.7)	7/523	1.3 (0.6–2.7)

*Abbreviations*: *MTCT* mother-to-child transmission, *CI* confidence interval, *LPV/r* lopinavir/ritonavir

^a^Reported for the 4609 pregnancies where infant HIV status was available

### Congenital abnormality

Among the live born infants with any prenatal exposure to LPV/r and data available (*n*=4609), including singleton and multiple births, an overall congenital abnormality rate of 2.9 % (95 % CI, 2.4–3.4 %) was observed, based on 134 infants with an identified birth defect (Table 5). No differences in the congenital abnormality rate were found when the results were stratified by infants who had any first-trimester exposure to LPV/r therapy (2.9 % [32/1116]; 95 % CI, 1.9–3.8 %) or were initially exposed during the second or third trimester (2.9 % [97/3403]; 95 % CI, 2.3–3.4 %). In stillborn infants, the overall congenital abnormality rate was 16.7 % (6/36; 95 % CI, 3.9–29.5 %).

**Table 5:** Congenital abnormalities in live born and stillborn infants (*n*=4645) by LPV/r exposure timing

	Overall	Any first trimester exposure to LPV/r	Earliest LPV/r exposure in second/third trimester
Total live births (singleton + multiple), *n*	4609	1116	3403
ᅟLive births with ≥1 abnormality, *n* (%)	134^a^ (2.9)	32 (2.9)	97 (2.9)
ᅟᅟ95 % CI	2.4–3.4	1.9–3.8	2.3–3.4
Stillbirths, *n*	36	8	27
ᅟStillbirths with ≥1 abnormality, *n* (%)	6 (16.7)	1 (12.5)	5 (18.5)
ᅟᅟ95 % CI	3.9–29.5	–	2.9–34.2
Category of abnormality			
ᅟCNS	8	3	5
ᅟEye, ear, face and neck^b^	4	0	4
ᅟCleft lip and/or palate	2	1	1
ᅟObstructive heart defects, right	2	0	2
ᅟObstructive heart defects, left	1	0	1
ᅟHeart—other defects	13	6	6
ᅟRespiratory system^c^	3	1	2
ᅟLower gastrointestinal system	8	2	6
ᅟFemale genitalia	1	1	0
ᅟMale genitalia^d^	6	1	5
ᅟRenal and urinary system^c^	13	2	10
ᅟLimb reduction/addition^e^	31	5	25
ᅟOther musculoskeletal defects	15	3	12
ᅟSkin and skin derivatives^f^	8	2	5
ᅟChromosome anomaly	15	4	11
ᅟOther organ systems-specified	4	1	3
ᅟSpecified syndromes	3	1	1
ᅟUnspecified abnormality	3	0	3

*Abbreviations*: *LPV/r* lopinavir/ritonavir, *CI* confidence interval, *CNS* central nervous system

^a^Includes 5 infants (5 live born) for whom information regarding the timing of LPV/r initiation was unavailable. None of these pregnancies were conceived on LPV/r

^b^4 minor

^c^1 minor

^d^3 minor

^e^28 minor

^f^7 minor

Among the 4645 live born and stillborn infants, the types of congenital abnormalities most often reported included limb reduction/additions (*n*=31), heart defects (*n*=16), musculoskeletal defects excluding limb reduction/additions (*n*=15), chromosome anomalies (*n*=15) and renal and urinary system defects (*n*=13).

## Discussion

The effectiveness of LPV/r-containing regimens during pregnancy was analysed using observational data from comprehensive national surveillance of 4864 pregnancies with LPV/r exposure in 4118 women with HIV infection in the United Kingdom and Ireland over a 10-years period. The results show that LPV/r-containing regimens during pregnancy were effective in maintaining or increasing maternal CD4+ T-cell counts, inducing or maintaining suppressed maternal viral loads and substantially reducing the risk of MTCT.

The low MTCT rates and the declining trend over time observed in this analysis are comparable with findings from previous analyses of NSHPC data [3, 4, 8], in which a steady decline in the MTCT rate over time has been documented, from a high of 18.5 % in 1990–1993 to 1.0 % in 2003–2006, and more recently to 0.46 % in 2010–2011. Similar improvements in MTCT rates have also been reported in other populations of women living in resource-rich areas during similar time periods [5–7].

The declining MTCT rates across Europe partly reflect the earlier use of ART in pregnancy among women not on treatment at conception, together with the increasing proportion of women who are already on suppressive regimens at conception. In this analysis, approximately one-fifth of pregnancies were conceived whilst the woman was receiving LPV/r-based ART, with an undetectable viral load in early pregnancy in 82 % of women. This is consistent with an earlier finding from a European cohort (women delivering in 2000–2011 in 9 Western European countries) where 19 % of women on ART at conception had a viral load of >200 copies/mL [30]. In the present analysis, practically all women with an undetectable viral load who conceived whilst on LPV/r-containing regimens maintained viral suppression throughout the pregnancy, whereas 69 % of women who initiated ART during pregnancy achieved an undetectable viral load by delivery.

In the absence of obstetric indications for a caesarean delivery, viral suppression before the end of pregnancy allows for a vaginal delivery [12]. In this analysis spanning from 2003 to 2012, slightly more than one-third of pregnancies were delivered vaginally, despite nearly three-quarters having an undetectable viral load by delivery. This finding may partly reflect the study period: since 2005, BHIVA guidelines have included planned vaginal delivery for women with suppressed viral load at term as an option and have recommended this approach since 2012 [12, 31]. However, recent findings from the NSHPC have also shown that women with undetectable viral loads delivering at maternity units with higher caseloads of women with HIV infection were significantly more likely to deliver vaginally than those delivering at less experienced units [32]; similar regional differences have also been observed in France [33].

Overall, nearly three-quarters of pregnancies were in women with delivery viral load below 50 copies/mL, and for most of the remainder, there was at least partial viral suppression (<1000 copies/mL). In France, approximately 78 % of women with HIV infection delivering in 1997–2010 had viral loads <400 copies/mL [33], and 76 % of a Spanish cohort delivering in 2004–2007 had an undetectable viral load (≤50 copies/mL) [5]. Factors associated with an increased probability of having a detectable viral load at delivery in a recent European pooled analysis included low CD4+ T-cell count (<200 cells/mm^3^), young maternal age, HIV diagnosis in the third trimester or intrapartum and injection drug use [15]. In the current analysis, among the small number of pregnancies with higher viral loads (≥1000 copies/mL) at delivery, ART was not initiated until the third trimester in 60 % of women compared with 18 % in the study population as a whole. MTCT rates were approximately 2 % for the mother-infant pairs where LPV/r therapy was not initiated until the third trimester, with no decline observed over time. Although only 14 % of mother-infant pairs overall were in this group, they accounted for 43 % of the MTCTs, highlighting the need to diagnose HIV infection and initiate ART earlier in some groups of women.

By 31 December 2014, 120 of the 517 previously indeterminate singletons were known to be uninfected, as well as another 7 twins or triplets. No new infections had been reported.

The current analysis of antenatal LPV/r exposure was associated with a similar incidence and rate of adverse pregnancy outcomes, including preterm delivery, low birth weights and incidence of congenital abnormalities as noted in other study populations. The congenital abnormality rate (2.9 %) in this infant population with antenatal exposure to LPV/r, regardless of overall exposure time, is similar to the rates previously reported for the entire NSHPC cohort and the uninfected general population from the region [34, 35]. Analysis of data from the Antiretroviral Pregnancy Registry (APR) has also not identified any relationship between birth defects and LPV/r use in 2458 pregnancies with exposure to LPV/r [36]. The rate of birth defects in the APR study population (2.2–2.4 %) was also comparable to the rate observed in the reference population.

Some studies have indicated an association between the use of protease inhibitor—containing ART during pregnancy and preterm delivery, whilst others have not [18, 22–28]. The preterm delivery rate in our study (12.7 %) is somewhat lower than reported in some earlier analyses of other European observational cohorts, although a number of studies have noted declines in preterm delivery rates in more recent years [15, 37]; these results may partly reflect the change in mode of delivery policy, leading to reduction of iatrogenic late preterm deliveries due to maternal HIV infection. The stillbirth rate here was 9.2 per 1000 infants, which is higher than the background rate in the United Kingdom (5.8 per 1000 infants in 2003 and 4.7 per 1000 infants in 2013) [38, 39], but slightly lower than the previously reported rate for the NSHPC as a whole from 1990 to 2006 (11.0 per 1000 infants) [4].

A strength of this study is that it includes data from a large unselected population of women and children with HIV infection collected through a long-term comprehensive active surveillance programme with high response rates. A potential limitation of the study, given that data are provided by antenatal care providers, is that early miscarriages and terminations are likely to be under-reported for women who did not receive care from an antenatal care clinic/provider early in their pregnancy. The difference in the proportion of miscarriages and terminations reported for women conceiving whilst on LPV/r and those initiating LPV/r in pregnancy is likely to be related to this limitation.

The low MTCT rates found in this study, which correlate with MTCT rates reported in other studies, suggest that the WHO goal of the elimination of vertical transmission of HIV in Europe [40] is achievable and within target for populations from resource-rich areas, although countries from Eastern Europe and Central Asia face additional barriers [9, 41]. Likewise, a review of 8 globally diverse studies that included a large proportion of patients from Africa noted MTCT rates of 0 to 2.8 % in women who received LPV/r therapy during pregnancy [29].

## Conclusions

Strategies for the management of HIV during pregnancy, including earlier initiation of ART during pregnancy, are the cornerstone for the prevention of MTCT. This analysis shows that a large number of pregnant women with HIV infection in the United Kingdom and Ireland have received LPV/r-containing ART regimens, and these real-world data demonstrate that these regimens are safe and effective for viral suppression during pregnancy and for protecting infants from perinatal HIV transmission.

## Availability of data and materials

All data on which the conclusions of this article rely are presented herein. All data processing was carried out at UCL Institute of Child Health, and no individual or identifiable patient data is available to third parties within our governance approvals. Regular aggregated data updates are freely available on the NSHPC website at www.ucl.ac.uk/nshpc, as are contact details for data requests.

## Abbreviations

3TC: lamivudine
ABC: abacavir
AIDS: acquired immunodeficiency syndrome
ART: antiretroviral therapy
BHIVA: British HIV Association
CD: cluster of differentiation
CI: confidence interval
FTC: emtricitabine
g: gram
HIV: human immunodeficiency virus
IDU: injecting drug use
IQR: interquartile range
LPV/r: lopinavir/ritonavir
mL: millilitre
mm: millimetre
MTCT: mother-to-child transmission
N/A: not available
NRTI: nucleoside reverse transcriptase inhibitor
NSHPC: National Study of HIV in Pregnancy and Childhood
RNA: ribonucleic acid
TDF: tenofovir
UK: United Kingdom
WHO: World Health Organization
ZDV: zidovudine
